# Management of diabetes mellitus and associated cardiovascular risk factors in Brazil – the Brazilian study on the practice of diabetes care

**DOI:** 10.1186/1758-5996-5-46

**Published:** 2013-08-26

**Authors:** Juarez R Braga, Alvaro Avezum, Sandra RG Ferreira, Adriana Forti

**Affiliations:** 1 Division of Cardiology, Peter Munk Cardiac Centre, University Health Network, Toronto General Hospital, Toronto, Canada; 2 Divisão de Pesquisa, Instituto Dante Pazzanese de Cardiologia, São Paulo, São Paulo, Brazil; 3 School of Public Health, University of São Paulo, São Paulo, Brazil; 4 Federal University of Ceará, Fortaleza, Brazil

**Keywords:** Diabetes mellitus, Risk factors, Cardiovascular disease

## Abstract

**Background:**

The Brazilian Study on the Practice of Diabetes Care main objective was to provide an epidemiological profile of individuals with type 1 and 2 diabetes mellitus (DM) in Brazil, concerning therapy and adherence to international guidelines in the medical practice.

**Methods:**

This observational, cross-sectional, multicenter study collected and analyzed data from individuals with type 1 and 2 DM attending public or private clinics in Brazil. Each investigator included the first 10 patients with type 2 DM who visited his/her office, and the first 5 patients with type 1 DM.

**Results:**

A total of 1,358 patients were analyzed; 375 (27.6%) had type 1 and 983 (72.4%) had type 2 DM. Most individuals were women, Caucasian, and private health care users. High prevalence rates of hypertension, dyslipidemia and central obesity were observed, particularly in type 2 DM. Only 7.3% and 5.1% of the individuals with types 1 and 2 DM, respectively, had optimal control of blood pressure, plasma glucose and lipids. The absence of hypertension and female sex were associated with better control of type 1 DM and other cardiovascular risk factors. In type 2 DM, older age was also associated with better control.

**Conclusions:**

Female sex, older age, and absence of hypertension were associated with better metabolic control. An optimal control of plasma glucose and other cardiovascular risk factors are obtained only in a minority of individuals with diabetes. Local numbers, compared to those from other countries are worse.

## Introduction

The prevalence of diabetes mellitus (DM) is increasing worldwide, associated with the aging population and the epidemiological transition [1]. Concomitant with the development of human societies which caused significant reductions in mortality related to infectious diseases, the adoption of a inadequate lifestyle with less physical activity and higher consumption of sugar and fat transformed obesity in a epidemic, which poses as a risk for the development of DM [2]. It is estimated that by 2030, 366 million individuals will be affected by the disease worldwide, being 11.3 million living in Brazil where this would represent an increase of almost three times the number of individuals with the disease today [3].

DM is currently the fifth leading cause of mortality worldwide by increasing the risk of cardiovascular disease when compared to populations without diabetes [4,5]. Besides that, microvascular complications markedly deteriorate their quality of life. Tight plasma glucose control, either with the use of insulin or oral glucose lowering agents, and the control of other cardiovascular risk factors, such as hypertension and dyslipidemia, are recommended nowadays in order to reduce morbidity and mortality associated with DM [6-14]. In addition to correcting glycemia, the combination of medications such as statins, antiplatelet agents and angiotensin-converting enzymes inhibitors is crucial in the treatment of patients affected by the disease.

Despite the evidence from relevant clinical trials, several studies conducted in Western countries have indicated a large gap between what is recommended and actually done in daily clinical practice [15-18]. The mere knowledge about efficacy of interventions does not revert in changes in clinical practice and combined efforts to improve patient care are required [19-21]. A initial step for the implementation of scientific knowledge involves an assessment of the current situation.

In Brazil, limited data regarding therapy and control of individuals with DM, as well as the frequencies of long-term complications and socioeconomic impact of the disease are available. The Brazilian Study on Practices of Diabetes Care (BDMPS) was designed to evaluate the therapy of type 1 and type 2 DM in the daily medical practice and how patients with diabetes are achieving the international recommendations for the control of plasma glucose and other cardiovascular risk factors.

The objective of the present study was to describe data of individuals with type 1 and type 2 DM, collected by the BDMPS in a standardized way, to provide an epidemiological profile of patients with diabetes mellitus in Brazil, concerning therapy and attendance to international guidelines in the medical practice.

## Methods

### Study design

The BDMPS is a cross-sectional, multicenter study, which collected and analyzed data from individuals with types 1 and 2 DM living in 3 out of 5 Brazilian regions.

### Study objectives

The primary objective was to evaluate the proportion of individuals with DM in Brazil with adequate control of glycated haemoglobin levels and other associated cardiovascular risk factors. The secondary objectives were to determine the factors associated to an adequate metabolic control; determine the therapy of patients with type 1 and 2 DM; evaluate the presence of comorbidities; and assess the impact of diabetes on hospitalizations and employment.

### Study population

Patients included were men and women, aged 18 years old and over, diagnosed with type 1 or 2 DM, accepting to participate in the study while visiting their doctor during the recruitment period. Those who were already enrolled in other clinical studies or who were on temporary use of insulin, such as for gestational diabetes, were excluded.

### Study procedures

Doctors with prior experience with insulin administration were selected at the discretion of the sponsor. They were trained before enrolling patients about the study procedures and application of the standardized questionnaires.

Throughout two weeks, each investigator included consecutively in the study, the first 10 patients with type 2 DM who visited his/her office, as well as the first 5 patients with type 1 DM.

During the inclusion visit, patients were questioned and evaluated for demographic profile, socioeconomic and cultural level, history of DM, associated cardiovascular risk factors, medications in use, laboratory measurements, outpatient services, screening for cardiovascular diseases, and hospitalization due to DM and its complications.

No test was performed specifically for the present study. Investigators relied on exams which have been ordered previously and were available for each patient according to their own clinical practice.

### Definitions

Hypertension was defined as a blood pressure ≥130/80 mmHg; dyslipidemia as LDL-cholesterol ≥100 mg/dL or HDL-cholesterol <40 mg/dL for men and <50 mg/dL for women or triglycerides ≥150 mg/dL; central obesity as waist circumference >90 cm in men and >80 cm in women. Adequate glycemic control was defined as a glycated haemoglobin ≤7%.

The outcome for the multivariable logistic regression model was the binary outcome “adequate metabolic control”. A patient was considered to be under adequate metabolic control when at least 5 of the following 8 variables were present: HbA1c ≤7%; blood pressure <130/80 mmHg; LDL-cholesterol <100 mg/dL; triglycerides <150 mg/dL; HDL ≥40 mg/dL for men or ≥50 mg/dL for women; abdominal circumference <80 cm in women or <90 cm in men; body mass index <25 kg/m^2^; and a never smoker.

### Statistical considerations

The sample size was calculated based on the objective of determining the utilization of insulin in patients with type 2 DM. Estimating the proportion of type 2 DM individuals in use of insulin equal to 17.5%, and considering an accuracy of 2% and a confidence interval of 95%, a sample of 1,385 participants would be necessary.

Data were expressed as means and standard deviations or the absolute number and percentages. The independent variables age, sex, level of education, human development index (HDI) and hypertension were chosen *a priori* based on clinical judgment as being associated with an adequate metabolic control. These variables were assessed in a multivariable logistic regression model and the results were expressed as adjusted odds ratios (OR) and 95% confidence intervals (CI). Age and HDI were treated as continuous variables and sex, level of education, and a diagnosis of hypertension as binary variables.

A *P* value <0.05 was considered to be significant for all tests. Statistical analysis was conducted using SAS statistical software (version 8.02) by the company Statistika Consultoria. This company was retained by the study sponsor. The sponsor submitted to monitoring ten percent of the total data obtained during the investigation, choosing randomly the investigators to be evaluated.

### Ethical considerations

All investigators obtained approval from their respective Research Ethics Committees. All participants signed the informed consent form before participation. There was no interference from the sponsor in the selection of the information to be used in the preparation of this manuscript.

## Results

### Characteristics of investigators

A total of 107 investigators from 3 out of 5 Brazilians regions participated in the study, 75 (70%) of which were endocrinologists. The average age of the investigators was 48 years, and the average time of professional practice was 23 years. The average number of patients seen each month by the investigators was 310 patients. Out of the 107 physicians, 71 (66.7%) attended mostly private patients.

### Characteristics of patients

A total of 1,430 individuals with DM were selected between June 1, 2007 and August 18, 2007, although only 1,358 were analyzed. The other 72 patients were excluded for not meeting the selection requirements or due to the absence of relevant data for qualifying (age below 18 years old, concomitant participation in a clinical trial, current temporary use of insulin, missing data about the type of DM, insulin use, and oral medications). Out of the 1,358 patients included in this analysis, 375 (27.6%) had type 1 DM and 983 (72.4%) had type 2 DM.

Table 1 shows the characteristics of the patients included according to the type of DM. As expected, individuals with type 1 DM were younger than those with type 2 DM. In both groups, there was a predominance of women, Caucasians, and private health care users. One third of the patients with type 2 DM and more than half of those with type 1 DM had post-secondary education.

**Table 1:** Characteristics of patients included

**Variables**	***Diabetes***	
	**Type 1 (N=375)**	**Type 2 (N=983)**
**Demographics**		
Age – yrs	34 ± 13.2	63 ± 11.3
Female gender	232 (61.9)	535 (55)
Caucasian	246 (65.6)	595 (61.1)
BMI – kg/m2	24 ± 3.7	29 ± 5.3
25-30	16 (4.3)	255 (26.5)
30-35	4 (1.1)	119 (12.4)
>35	12 (3.2)	3 (0.3)
Waist circumference, male, cm	86 ± 11.4	102 ± 12.8
Waist circumference, female, cm	80 ± 11.9	96 ± 13.8
**Socioeconomic profile**		
University level or higher	201 (53.7)	321 (33.5)
Private health insurance	310 (82.7)	833 (85.1)
Full/part time employment	263 (70.7)	381 (38.9)
Sick leave if employed*	26 (6.9)	21 (2.1)
Unemployment related to disability	67 (17.8)	364 (37)
**Laboratorial measurements**		
Fasting plasma glucose – mg/dL	151 ± 81.7	143 ± 57.2
Glycated hemoglobin – %	8.4 ± 1.9	7.5 ± 1.6
Total cholesterol – mg/dL	179 ± 45.2	181 ± 41.6
LDL-cholesterol – mg/dL	102 ± 33.5	102 ± 39.2
HDL-cholesterol – mg/dL	58 ± 41.2	48 ± 22.5
Triglycerides – mg/dL	103 ±74.9	157 ± 105
**Time since diagnosis** – yrs	16 ± 10.8	11 ± 8.7
**Medications**		
Use of glucose-lowering drug	43 (11.5)	827 (84.1)
Metformin	31 (8.3)	270 (27.5)
Sulphonylureas	1 (0.3)	60 (6.1)
Metformin + sulphonylureas	1 (0.3)	309 (31.4)
Others	10 (2.7)	188 (19.1)
Number of glucose-lowering drugs		
1	37 (9.9)	379 (38.6)
2	6 (1.6)	344 (35)
>2	0 (0)	104 (10.6)
Insulin	375 (100)	343 (34.8)
ACEi/ARB	85 (22.7)	582 (59.2)
Antiplatelet agent	66 (17.6)	465 (47.3)
Statins	68 (18.1)	478 (48.6)
Hospital admissions*	30 (9.1)	40 (4.5)
**Diabetes complications**		
Retinopathy	106 (28.3)	204 (20.7)
Neuropathy	65 (17.3)	200 (20.3)
Proteinuria¶	89 (23.7)	190 (19.3)
Dialysis	16 (4.3)	43 (4.3)
Acute coronary syndrome	7 (1.8)	102 (10.4)
Heart failure	5 (1.3)	50 (5)
Stroke	4 (1)	42 (4.2)
Peripheral vascular disease	14 (3.7)	134 (13.6)

Plus – minus values are means ± standard deviations. Numbers in brackets mean percentage of an absolute number. *N* number, *yrs* age in years, *BMI* body mass index, *ACEi* angiotensin converting enzyme inhibitor, *ARB* angiotensin receptor blocker.

*: related to the past 3 months of the visit.

¶: Proteinuria was defined as raised urine protein:creatinine ratio (>200 mg/g) or urine albumin:creatinine ratio (>250 mg/g for men and >355 mg/g for women) or total protein 24-hour excretion (>300 mg/day).

In the previous 3 months before the baseline visit, 9.1% of type 1 DM patients and 4.5% of type 2 DM ones had been admitted to a hospital. In relation to the complications associated with DM, nearly 40% of individuals with type 2 DM were on sick leave, along with almost 20% of those with type 1 DM.

### Treatment characteristics

Eighty-four percent of patients with type 2 DM were in use of a glucose-lowering drug; 45% were is use of at least 2 drugs. The use of angiotensin-converting enzyme inhibitors and angiotensin receptor blockers, antiplatelet agents, and statins were 22.7% and 59.2%; 17.6% and 47.3%; 18.1% and 48.6% in type 1 and type 2 DM, respectively.

### Complications associated with diabetes

Table 1 includes the frequency of micro and macrovascular complications associated with diabetes. Prior acute coronary syndrome was reported in 1.8% and 10.4% of the type 1 and 2 DM, respectively, and cerebrovascular accident in 1% and 4.2%.

### Associated cardiovascular risk factors

Hypertension, dyslipidemia, and central obesity were present in 23%, 28.5%, and 39% of type 1 DM and 75.7%, 71%, and 84% of type 2 DM patients (Table 2). Table 3 shows the proportion of patients with adequate control of blood glucose and other cardiovascular risk factors. Adequate glycemic control of at least 5 cardiovascular risk factors was found in 35.7% of type 1 and 14.4% in patients with type 2 diabetes. Only 16.7% of the individuals with type 2 DM had blood pressure below 130/80 mm Hg, and around 40% had the LDL-cholesterol under 100 mg/dL (Table 3).

**Table 2:** Prevalence of cardiovascular risk factors

	***Diabetes***	
	**Type 1 (N=375)**	**Type 2 (N=983)**
**Hypertension**	84 (22.8)	729 (75.7)
**Dyslipidemia**	103 (28.5)	672 (70.9)
**Smoking**		
Current	42 (11.4)	107 (11.3)
Previous	21 (5.7)	160 (16.9)
Never	305 (82.9)	678 (71.7)
**Central obesity**	146 (38.9)	822 (83.6)
**Metabolic syndrome**		
NCEP	-	562 (57.1)
IDF	-	522 (53.1)

Numbers in brackets mean percentage of an absolute number.

*NCEP* National Cholesterol Education Program criteria, *IDF* International Diabetes Federation criteria. Central obesity defined as waist circumference >90 cm in men and >80 cm in women according to the IDF recommendations for South Americans.

**Table 3:** Proportion of individuals with adequate metabolic control according to the levels of glycated hemoglobin, blood pressure, lipids, waist circumference, BMI, and smoking

	***Diabetes***	
	**Type 1 (N=375)**	**Type 2 (N=983)**
Blood pressure <130/80 mm Hg	162 (43.3)	162 (16.7)
Glycated hemoglobin <7%	77 (22.1)	379 (43.2)
LDL-cholesterol <100 mg/dL	138 (36.8)	420 (42.7)
TGC <150 mg/dL	248 (66.1)	507 (51.5)
HDL-cholesterol (≥40 mg/dL, males)*	81 (77.9)	218 (56.0)
HDL-cholesterol (≥50 mg/dL, females)*	129 (75.0)	218 (47.4)
Waist circumference (<90 cm, males)*	98 (70)	76 (17.4)
Waist circumference (<80 cm, females)*	123 (54.2)	70 (13.2)
BMI <25 kg/m^2^	261 (69.2)	218 (22.1)
Non-smokers	305 (82.9)	678 (71.7)
Adequate control of at least 5 risk factors	134 (35.7)	142 (14.4)

Numbers in brackets mean percentage of an absolute number.

*TGC* tryglicerides, *BMI* body mass index.

* To calculate the proportion of individuals with normal waist circumference and HDL-cholesterol among type 1 and 2 DM individuals according to sex, we used the total number of females or males present in each group on the denominator.

The multivariable logistic regression analysis showed that female gender and absence of hypertension were independently associated with adequate control of cardiovascular risk factors in type 1 DM. In type 2 DM, in addition to those variables, older age was also associated with adequate metabolic control (Table 4).

**Table 4:** Variables associated to an adequate metabolic control

	***Diabetes***					
	**Type 1 (N=241)**			**Type 2 (N= 698)**		
	**OR**	**95% CI**	**P value**	**OR**	**95% CI**	**P value**
**Female gender, (yes vs. no)**	1.61	1.21-2.15	0.001	1.22	1.004-1.5	0.046
**Age, per 1 year**	0.99	0.97-1.02	0.581	1.02	1.001-1.04	0.04
**Hypertension, (no vs. yes)**	2.22	1.51-3.26	<.0001	1.48	1.19-1.85	0.0005
**HDI, per 1 point**	5.9	0.32-107.8	0.45	12.3	0.78-194.4	0.36
**Higher education, (yes vs. no)**	0.94	0.71-1.24	0.67	0.97	0.79-1.19	0.74

*OR* odds ratio, *CI* confidence interval, *N* number, *HDI* human development index.

## Discussion

Physicians who participated in this study were above the national average age, which is made up of almost 65% under the age of 40 years. Furthermore, almost half the practicing doctors in Brazil graduated less than 15 years ago. Also, the specialist title in endocrinology by the majority of investigators suggests that the participating doctors should have a good experience in the medical practice [22].

Many studies have demonstrated a linear association between the levels of glycated haemoglobin (HbA1c) and micro and macrovascular complications - the higher the blood glucose levels over time, the greater the association with the development of changes associated with DM [23].

Conversely, intensive control of blood glucose levels, and the achievement of reductions in the levels of glycated haemoglobin prevents macro and microvascular complications in type 1 and type 2 DM [24].

However, our data shows that most patients do not reach adequate glycemic control. Twenty-two percent and 43.2% of those individuals with type 1 and 2 DM had HbA1c below 7%, respectively. In a previous survey of 6,671 patients with diabetes from public hospitals in Brazil, the prevalence of inadequate glycemic control (HbA1c ≥7.0%) was 76% [25]. According to the type of DM, inadequate glycemic control was identified in 90% of type 1 and in 73% of type 2 DM [25]. This difference in the numbers suggests that the type of health care (public *versus* private care) may have important consequences over metabolic control.

The individuals studied, especially those with type 2 DM, had a high prevalence of other cardiovascular risk factors, such as hypertension, dyslipidemia, and central obesity, under inadequate control.

A study analyzing the results from the National Health and Nutrition Examination Survey (NHANES) showed that the Brazilian figures are way inferior than those found in the United States. In the American study, 36% of the individuals examined had blood pressure below 130/80 mm Hg, and 7.3% had optimal control of hypertension, dyslipidemia and glucose [17]. In a similar study conducted in France, 37% of patients with type 2 DM had blood pressure below 130/80 mm Hg, and 28% had adequate control of plasma lipids [18].

Our study has some strength. First, we considered not only the glycemic control but also other metabolic abnormalities responsible to the increased cardiovascular risk in individuals with diabetes. Second, by using established definitions for diabetes control and other cardiovascular risk factors, we were able to put the Brazilian numbers in perspective to the international literature and assess the local practice. Third, the selection of investigators, although not random, covered the main Brazilian regions, providing a national picture of diabetes care.

The present study has several limitations. Diabetes mellitus types 1 and 2 are quite different conditions. Although characterized by increased glycemic levels, it may be improper to compare individuals with diseases quite distinct. There was no standardization of laboratory results, as each investigator used the locally available laboratory, although no significant changes were expected in the results obtained, had they been done in a central laboratory. As the selection of investigators was based on ability and experience in the insulin therapy and layouts of high complexity, this may have reflected in a greater representation in the study sample of individuals with long-term evolution of diabetes, greater difficulty of control, and therefore a higher prevalence of complications in connection with the disease. Although, the study protocol determined that investigators were supposed to enroll consecutive patients, we cannot disregard the possibility of selection bias.

Most patients included were private health care users with high levels of education. Therefore, they do not represent the overall Brazilian population with DM. This limits the generalizability of our findings. However it is possible that for the average individual with DM in Brazil, the access to less-experienced physicians, an inferior educational level, and limited availability of diagnostic tests and more advanced medications, results could be much worse than those obtained in the present study.

The projections of DM for the next decades indicate marked increase in cases of cases along with microvascular and macrovascular complications. Management of the disease should attempt to modify different aspects of an individual’s life, such as eating habits, physical activity levels, correct use of multiple medications and insulin administration, laboratory monitoring, and screening for cardiovascular diseases.

There are consistent evidences that optimal glycemic control, along with control of hypertension, dyslipidemia, smoking cessation, and weight loss are necessary for reducing cardiovascular risk in these patients. However, even in a favourable environment in which patients with high educational level have access guaranteed by private insurance to experienced physicians, only a small number achieve proper metabolic control. Comparing it to international reports, the Brazilian figures are poorer, with worse levels of control of plasma glucose levels and associated cardiovascular risk factors.

We conclude that the BDMPS is relevant to disclose the scenario of the DM control in an emerging country like Brazil, and calls attention to the need of better educational strategies to try improve this picture.

## Competing interest

The authors, coordinators, and investigators have been compensated by the sponsor to conduct this study.

## Authors’ contributions

JB was responsible for data analysis, data interpretation, and writing the manuscript; AA was responsible for conception, design, data interpretation, and writing the manuscript; SF was responsible for data interpretation, and writing the manuscript; AF was responsible for data interpretation, and writing the manuscript. All authors read and approved the final manuscript.

## The following members assisted in the coordination of the study

Álvaro Avezum, Hélio Penna Guimarães, Patrícia Smith, and Lígia Nasi Laranjeira.

## The following investigators participated in the study (listed in alphabetical order)

Adriana Estevam Moretti, Alessandra Uchôa Massad, Álvaro Avezum, Ana Chartuni Teixeira Cury, Ana Claudia Teixeira Jacob Machado, Ana Elizabeth Cabral de Menezes Marinho, Anete Hannud Abdo, Ângela Regina Nasário Sabbag, Aniete Carolina Roma Castro, Antônio Braojos Dantas, Antônio Carlos Lerario, Antônio Tarcísio de Faria Freire, Arnaldo Alves de Mendonça, Bernardo Leo Wajchenberg, Bruno Geloneze Neto, Carlos Frederico Campos, Carlos Gun, Carlos Roberto Netto de Menezes, Carmem Alice Coelho Rezende, Célia Aparecida Uliani Zimmermann, Charles Rodrigues, Christina Lourdes Porto Gaspar Moreira Muniz, Claudia Cozer Leite, Cláudio Guimarães Bernardo, Cleber Rinaldo Favaro, Cristina Figueiredo Sampaio Façanha, Dario Cezar de Vasconcellos, Davis Taublib, Denise Maria Loyola Zolet, Denise Reis Franco, Diógenes Bernardes, Edgar D’Ávila Niclewicz, Edília Maria Ribeiro Dias, Edson da Cunha, Edson Perrotti Santos, Elcio Gomes Junior, Emmanuel de Abreu, Enecy Maria Laborne Brenlha, Erico Paulo Heilbrun, Ernestina Auache, Flávia Ozório Pereira, Francisco José da Cunha Stamato, Freddy Goldenberg Eliaschewitz, Gladys de Jesus Matta, Gláucia Moraes Bastos, Helena Trindade Camargo, Honomar Ferreira de Souza, Ivan Arbex, Ivan dos Santos Ferraz, João Eduardo Nunes Salles, João Paulo Iazigi, José Carlos Aydar Ayoub, José Henrique de Almeida Netto, José Marcio Ribeiro, José Otávio Alquezar Gozzano, José Tasca, Josivan Gomes de Lima, Jucinéia Aparecida de Oliveira, Julio Henrique Bom, Laerte Ferreira Damasceno, Leda Aparecida Daud Lotaif, Levimar Rocha Araújo, Lúcia Helena Silva, Luciano Ricardo Giacaglia, Lucy Mara Gomes Baetas Valente, Luis Cuadrado Martin, Luiz Guilherme Lopes Junior, Marcelo Alexandrino da Silva, Marcelo Eduardo Peralta, Márcia Jablonka Kelman, Márcio Barbosa Guimarães Cota, Márcio Correa Mancini, Marcos André Malta Dantas, Marcos Antonio Lopes, Marcos Antônio Tambascia, Maria da Conceição Carneiro Mamone, Maria Edna de Melo, Maria Isabel Guimarães Favaro, Maria Terezinha Infantosi Vannucchi, Maria Victoria Martinez Descalzo, Mariana Narbot Ermetice, Mariângela Occhioso, Milton Elmor Filho, Mônica Maria Teixeira, Patrícia dos Santos Moniz Martins, Patrícia Smith, Patrícia Teófilo Monteagudo, Paula Fernandes, Paulo de Tarso Freitas, Priscila Lustoza Gomes Sampaio, Rejane Bisacchi Coelho Corrêa de Oliveira, Ricardo Cairo de Camargo, Ricardo da Silva Sá, Roberto Jorge da Silva Franco, Rosangela Maria de Almeida, Rosângela Roginski Réa, Rubens Antunes da Cruz Filho, Samuel Cunha, Sandra Lucia Minanni, Sidney Senhorini, Silvana Maria C P de Souza Queiroz, Silvio Hock de Paffer Filho, Simone Siqueira Meirelles, Tadeu Alencar, Tereza Maria Figueredo Campos Fontes, Vladimir Gomes Oliveira and Walter José Minicucci.
